# Precise timing when hitting falling balls

**DOI:** 10.3389/fnhum.2014.00342

**Published:** 2014-05-22

**Authors:** Eli Brenner, Ben Driesen, Jeroen B. J. Smeets

**Affiliations:** Faculty of Human Movement Sciences, MOVE Research Institute, VU University AmsterdamNetherlands

**Keywords:** interception, timing, hitting, gravity, motor control, precision, baseball, vision

## Abstract

People are extremely good at hitting falling balls with a baseball bat. Despite the ball's constant acceleration, they have been reported to time hits with a standard deviation of only about 7 ms. To examine how people achieve such precision, we compared performance when there were no added restrictions, with performance when looking with one eye, when vision was blurred, and when various parts of the ball's trajectory were hidden from view. We also examined how the size of the ball and varying the height from which it was dropped influenced temporal precision. Temporal precision did not become worse when vision was blurred, when the ball was smaller, or when balls falling from different heights were randomly interleaved. The disadvantage of closing one eye did not exceed expectations from removing one of two independent estimates. Precision was higher for slower balls, but only if the ball being slower meant that one saw it longer before the hit. It was particularly important to see the ball while swinging the bat. Together, these findings suggest that people time their hits so precisely by using the changing elevation throughout the swing to adjust the bat's movement to that of the ball.

## Introduction

People are extremely good at intercepting a falling ball with a bat (McLeod et al., 1985; Brenner et al., 2012). They can time their attempts to hit a ball with a precision of about 7 ms (we use the standard deviation as our measure of temporal precision throughout this article). This is much better than the temporal precision in indicating which of two targets stopped moving first (27 ms at best; Figure 3C of Tadin et al., 2010) or changed length first (35 ms at best; Figure 5 of Baruch et al., 2013). It is also much better than the precision in indicating whether the interval between the first and the second of three flashes was longer or shorter than the interval between the second and the third flash (about 30 ms; Figure 2A of Zanker and Harris, 2002). The precision with which movements of the two hands can be synchronized is about 14 ms (Figure 4E of Doumas and Wing, 2007; Figure 6D of Doumas et al., 2008), as is the precision with which expert pianists can time their keystrokes (Figure 3B of Goebl and Palmer, 2013). To our knowledge, temporal precision is only less than 7 ms for judging which of two adjacent targets was flashed first (Figure 1 of Westheimer and McKee, 1977a), in which case the temporal order is presumably judged from the perceived motion (Brenner and Smeets, 2010).

Moreover, there is abundant evidence that the human visual system is quite poor at judging the instantaneous acceleration (Gottsdanker et al., 1961; Werkhoven et al., 1992), yet the above-mentioned high temporal precision of interception is achieved with a falling ball that is accelerated by gravity. Thus, people must be relying on their experience with previous balls or with falling objects in general to judge the acceleration (Zago et al., 2004, 2009), or continuously be adjusting their movements to minimize the influence of misjudging the acceleration (Lee et al., 1983). Altogether, the temporal precision in intercepting falling balls appears to be at the very limit of what one could expect considering the required visual judgments about the ball's approach and the need to move the bat accordingly.

Not all interception studies report a temporal precision of about 7 ms. Poorer precision has been found when hitting falling balls under more constrained conditions (23 ms at best; Table 1 of Katsumata and Russell, 2012), when hitting virtual targets that move at a constant velocity (22 ms; Table 2 of Brenner and Smeets, 2009), and when hitting real targets that move at a constant velocity (12.5 ms at best; data including misses in Figure 4 of Tresilian et al., 2003). Moreover, a high precision appears to require continuous updating of sensory information (Bootsma and van Wieringen, 1990; Land and McLeod, 2000; Brenner and Smeets, 2011).

McLeod and Jenkins (1991) argued that the temporal precision in batting a (falling) ball is limited by the spatial resolution of the human eye. They did so on the basis of calculations involving the rate of expansion of the ball's retinal image and their estimate of the latencies involved in guiding the hitting movement. Michaels et al. (2001) observed differences between monocular and binocular performance when intercepting a falling ball, suggesting that retinal image size may not be the only relevant information. For catching, Rushton and Wann (1999) proposed that information based on retinal image size is combined with binocular information to improve performance (also see Regan, 1997; van der Kamp et al., 1997; Bennett et al., 2000; Regan and Gray, 2000). In a similar way, we here consider that the ball's changing elevation angle might provide critical information, because the ball does not fall straight toward the eye (since it is hit at some distance from the body).

In one of the previous studies in which a temporal precision of about 7 ms was found (Brenner et al., 2012), balls were released from a height of 5.7 m and were hit about 1.24 m above the ground. Three parameters change smoothly as the ball approaches: the ball's angular size and the binocular convergence that is required to keep looking at a position on the ball (binocular disparity) increase until the ball passes the batsman's eyes, while the ball's elevation angle continuously decreases. Assuming that the ball (6.6 cm diameter) passed about 0.7 m from the batsman's eyes, and that the eyes were 1.6 m above the ground with an inter-pupil distance of 6.6 cm, we can calculate that 200 ms before the ball is hit, the diameter of its image is increasing at 12°/s. The binocular disparity at that time is also changing at 12°/s, and the elevation angle is changing at 235°/s. Similarly, 100 ms before the ball is hit, its image size and binocular disparity are changing at 24°/s and its elevation angle at 349°/s. These calculations are based on a ball (0.057 kg) falling under gravity (9.81 m/s^2^) with air resistance [drag force = ½ρ *v*^2^*C*_*D*_*A*, using 1.225 for the density of air (ρ), 0.6 for the drag coefficient (*C_D_*; based on Goodwill et al., 2004) and 0.0034 m^2^ for the ball's cross-sectional area (*A*). *v* is the ball's speed]. The calculations were verified by comparing the calculated speed of the ball with the measured speed near the height at which the ball was hit.

Although the calculations show that the rate of change is an order of magnitude larger for the elevation angle than for the other two parameters, one must keep in mind that in terms of angles, the precision with which people can judge changes in image size is presumably much higher than the precision with which they can judge changes in elevation or ocular convergence. The former is probably limited by the retinal resolution (about 1′ arc in the fovea, although higher precision can be achieved in some tasks; Westheimer and McKee, 1977b) whereas the latter are probably limited by the resolution of judging eye orientation (about 6′ arc at best; Brenner and Smeets, 2000). We therefore started the current study, in which people tried to hit falling balls with a bat (as in Brenner et al., 2012), by varying the circumstances in ways that are likely to affect the above-mentioned sources of information. In subsequent experiments we examined how various other manipulations influence people's timing when hitting falling balls.

In the first experiment, we evaluated the role of binocular information by comparing how well subjects hit tennis balls with one eye closed, with how well they did so when they had both eyes open. We evaluated the importance of a high resolution with which to detect changes in the ball's retinal image size in two ways: by having subjects wear reading glasses that blurred the images on their retinae, and by reducing the ball's retinal image size by having subjects hit smaller balls. The manipulations hardly affected the subjects' performance. In the first experiment the ball always appeared at the same height, moving at the same speed. In the second experiment, we varied the ball's speed at the time it appeared, to check that people were not just hitting a fixed time after the ball appeared, and more generally to evaluate to what extent people were relying on feedback from previous trials. Whether all balls were moving at the same speed or not made no difference, but slower balls were hit more precisely. In the third experiment, we examined whether the fact that people's timing was less precise when the ball moved faster was because of the speed itself, or because a faster ball had to be hit sooner after it had appeared. We found that it was clearly the time that the ball was visible, and not its speed, that determined the temporal precision. In the second and third experiments, increasing the time that the ball was visible meant that one saw it longer before the hit. In the fourth experiment, we examined whether seeing the ball earlier was particularly important because it allowed one to better initiate the hit.

## Methods

**Figure 1:**
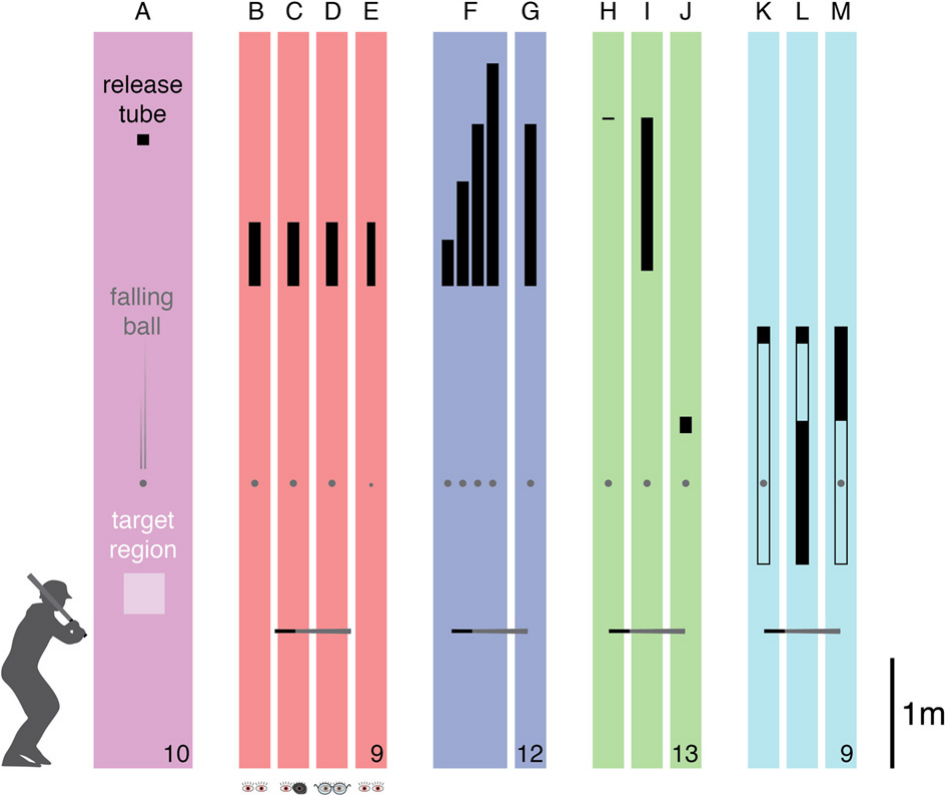
**Overview of task and conditions**. Subjects had to try to hit a falling ball toward a target region. The ball was released through a tube (indicated in black). **(A)** The configuration of the corresponding condition in Brenner et al. (2012). **(B–E)** The four conditions of Experiment 1: *baseline*, *monocular*, *blurred*, and *small ball*. **(F,G)** The two conditions of Experiment 2: *varying speed* and *fixed speed*. (**H–J**) The three conditions of Experiment 3: *fast*, *fast & short*, and *slow & short*. **(K–M)** The three conditions of Experiment 4: *transparent tube*, *early vision*, and *late vision*. The drawings are approximately to scale, showing the lengths and heights of the release tubes as well as the approximate height of the horizontal “bat” at the anticipated moment of impact. The gray disk represents the ball at an arbitrary moment. The number of participants that were included in the analysis is indicated at the bottom right for each experiment.

The task was always to hit a falling ball with a bat. The subject's aim was for the ball to hit a target that was at waist height, several meters away (shown schematically in Figure 1A). Except when mentioned otherwise, the balls were regular sized tennis balls. The bat was a children's foam-covered baseball bat that we bought in a toy shop (total length: 68.5 cm; diameter of relevant section: 5 cm). The experiments were conducted in a well-lit sports hall within our department. The balls were released from various heights and fell through tubes of various lengths, allowing us to independently vary the balls' speeds and the times for which they were visible before being hit. The subjects were all young adults. None of the subjects were aware of the hypotheses, but the manipulations were quite evident. Subjects were instructed to stand and hit in such a manner that the bat would be oriented approximately horizontally when it hit the ball, but received no further instructions about how to perform the task. The study is part of a research program that has been approved by the local ethical committee, and all subjects signed the standard informed consent form. All tested conditions and exclusions are reported.

### Experiment 1

Eleven male, right-handed subjects (with normal binocular vision as tested with the “Stereo fly test”) agreed to participate in Experiment 1, but two subjects' data were not analyzed. In one case this was due to equipment failure. In the other it was because we only noticed half way through the session that the bat was not oriented approximately horizontally at the time of the hit, which made it impossible to reliably estimate the timing precision. We will henceforth only consider the remaining 9 subjects.

There were four conditions (Figures 1B–E) that were performed in separate blocks of trials. The order in which the conditions were presented was selected at random for each subject. There were breaks both between and half way through the blocks, while the experimenters gathered the balls. Each block of 60 trials was preceded by 12 practice trials that were not analyzed. In one condition (*baseline*) subjects hit the tennis balls with no additional restrictions. In a second condition (*monocular*) they kept one eye closed when doing. They were free to choose which eye to close (four closed their right eye and five closed their left eye). In a third condition (*blurred*) two near-sighted subjects who normally wore spectacles (−2.5 and −4.5 D) removed their spectacles and the others wore +2.5 D reading glasses (making them near-sighted, so that the ball's retinal image will have been blurred, especially early during its fall). In the fourth condition (*small ball*) the tennis balls (diameter of 6.6 cm) were replaced by bouncing balls (diameter of 3.6 cm). Calculations suggest that the latter were not only smaller but were also moving almost 3% faster than the tennis balls at the time of the hit.

The balls were released from a height of 4.9 m. The release tube was 58 cm long, and had a diameter of 7 cm for the tennis ball and of 5 cm for the bouncing ball. The target was at a distance of 5.6 m. Except for scoring whether or not the bat touched the ball, we also used an Optotrak 3020 system (Northern Digital Inc., Waterloo, Ontario, Canada) to measure the movement of an infrared light emitting diode that was attached to the tip of the bat (positions determined at 800 Hz). For each subject and condition we determined the fraction of balls that were “touched” by the bat. We used the measured horizontal and vertical speeds of the tip of the bat, and the curvature of the tip of the bat's path (determined during the time at which it moved faster than 4 m/s, assuming a constant curvature), to estimate the speed of the relevant part of the bat (the part with which the ball is hit) at the time at which the bat hit or passed the ball. In doing so we assumed that the relevant part of the bat was 20 cm from the tip, and that the bat's movement was a rotation around a point along an extension of the bat's main axis (so this point can be derived from the measured curvature). For trials in which the ball was hit, we also measured the direction of the acceleration of the bat caused by the impact with the ball.

We need to know the vertical velocity of the ball (relative to the relevant part of the bat) and the horizontal velocity of the relevant part of the bat in order to estimate the time window for hitting the ball. Knowing the time window and the fraction of touched balls, and assuming a normal distribution of timing errors, would be enough to estimate the temporal precision if we could be sure that subjects' average timing was correctly chosen so as to touch as many balls as possible with the bat. However, the subjects' aim was not to touch as many balls as possible, but to hit the target with the balls, so we also used information about the direction of the impact between the bat and the ball to estimate the average timing.

We are primarily interested in the standard deviation of the temporal errors (σ). We assume that these errors are normally distributed, but consider the possibility of a bias (μ) to arrive a bit later than would be optimal for touching as many balls as possible, because the participants' task was not to touch the balls but to hit them toward a target. Thus, the probability of arriving at time *t* with respect to the time for which the bat would touch the most balls is:

$$P(t)=\frac{1}{\sigma \sqrt{2\pi }}e^{-\frac{{\left(t\;-\;\mu \right)}^{2}}{2\sigma^{2}}}$$

The range of times for which the bat will touch the ball and the direction in which the bat will hit the ball for a given time *t* depends on the horizontal speed of the bat (*V_bat_*) and the vertical speed of the ball relative to the bat (*V_ball_*). For a sum of the radii of the bat and the ball of *r*, the ball will be hit if the value *h(t)* is positive:

$$h(t)=V_{bat}^{2}t^{2}-\;\left(V_{bat}^{2}+\;V_{ball}^{2}\right)\left(V_{bat}^{2}t^{2}-\;r^{2}\right)$$

The direction of the acceleration at impact (assuming a perfectly elastic collision) is then:

$$d(t)=\arctan\text{ ​}\left(\frac{V_{ball}\text{​}\left(\sqrt{h(t)}+V_{bat}^{2}t\right)}{V_{bat}\text{​}\left(\sqrt{h(t)}-V_{ball}^{2}t\right)}\right)$$

In order to estimate σ from the number of balls that were hit (*n_hit_*) and the mean measured direction of the acceleration of the bat at impact (*d*_*m*_) we solve:

$$\int_{h(t)\;>\;0}p(t)dt=\frac{n_{hit}}{n_{total}}$$

and

$$\int_{h(t)\;>\;0}p(t)d(t)dt={\bar{d}}_{m}$$

where *n_total_* is the total number of balls presented. We solved these two integrals simultaneously for each subject and condition to estimate σ (and μ). We compare the timing precision (σ) across the conditions with a repeated measures analysis of variance.

### Experiment 2

Six male and six female subjects (one left-handed) participated in Experiment 2. There were two conditions (Figures 1F,G) that were performed in separate blocks of trials in a counterbalanced order. Again, there were breaks both between and half way through the blocks while the experimenters gathered the balls. Each block of 100 trials was preceded by 12 practice trials that were not analyzed. In one condition (*varying speed*) 40 balls fell from a height of 5.79 m, and 20 balls each from heights of 4.74, 5.27, and 6.34 m, so they were moving at different speeds at the time that they were to be hit. The 100 trials were presented in a random order, so that the different speeds were interleaved. In a second condition (*fixed speed*) all 100 balls fell from a height of 5.79 m. In all cases the ball came into view (exited the release tube) at a height of 4.32 m. The target was at a distance of about 5 m.

In this experiment we filmed the hits at a high temporal resolution (1000 Hz) and low spatial resolution (224 by 64 pixels) with a Casio Exilim EX-ZR1000 camera. To compensate for the low spatial resolution we zoomed in on the region in which we expected the ball to be hit. As a result, data were lost if subjects hit a ball much higher or lower than expected. A calibration panel that was placed in the ball's path before the experiment allowed us to convert pixels in the image into distances in the world. The camera's frequency was verified by filming a rapidly flashing light emitting diode of which the frequency was determined with an oscilloscope. The camera was about 4 m from the ball's path, orthogonal to the direction toward the target, so that at the time of the hit the ball was moving downwards in the image, in front of the subject, and the tip of the bat was more or less facing the camera, moving to the right in the image for the right-handed subjects, and to the left for the left-handed subject (in which case the subject and camera switched sides with respect to the ball's path).

The timing of the bat with respect to the ball was determined by stepping through the recordings while counting the images. For each trial we determined the difference in time between when the ball and the bat reach the point at which their paths cross. If the ball was hit before reaching that point, the spatial calibration was used to calculate the time at which the ball would have crossed that point. The precision is the standard deviation of these time differences. We compared the timing precision for the balls that fell from a height of 5.79 m across the two conditions with a repeated measures analysis of variance. We compared the timing precision for the balls that fell from the four different heights in the *varying speed* condition with a second repeated measures analysis of variance. We used *t*-tests with Bonferroni correction to identify the heights for which the subjects had significantly different precision.

### Experiment 3

Nine male and five female subjects (one left-handed) participated in Experiment 3. One female subject's data was not included in the analysis, because a majority of her hits took place outside the image. There were three conditions (Figures 1H–J) that were performed in separate blocks of trials. The order in which the conditions were presented was selected at random for each subject. Each block of 60 trials was preceded by 4 practice trials that were not analyzed. There were breaks between the blocks while the experimenters gathered the balls.

In one condition (*fast*) the balls fell from a height of 5.85 m, so they were visible for almost a second before they reached a height of 125 cm (the height at which we anticipated that the ball would be hit). In a second condition (*fast & short*) the ball fell from the same height, but during the first 1.39 m it was within a release tube, so it was moving at the same speed but had only been visible for 450 ms when it reached a height of 125 cm. In a third condition (*slow & short*) the ball was released at a height of 3.13 m, with a 15 cm release tube, so it moved more slowly but was also visible for 450 ms by the time it reached a height of 125 cm.

The target was at a distance of about 4.4 m and the Casio Exilim EX-ZR1000 camera at a distance of about 3.2 m from the ball's path. For trials in which the ball was hit, estimates of the timing of the hit were refined by also measuring the direction in which the ball moved after the hit. Otherwise the procedure was identical to that of Experiment 2. We compared the timing precision across the conditions with a repeated measures analysis of variance, and then used *t*-tests with Bonferroni correction to identify the conditions in which the subjects had significantly different precision.

### Experiment 4

Seven male and two female subjects (one left-handed) participated in Experiment 4. In this experiment the ball always fell from a height of 3.95 m, first falling 15 cm through an opaque release tube, and then 200 cm through a (also 7 cm diameter) transparent tube, which it exited at a height of 1.8 m. The ball's motion in this experiment was not precisely as described by the equation given in the introduction, because the balls clearly moved differently within the tube (probably due to the larger air resistance), so the positions at which the tube had to be covered to achieve the desired viewing times were based on measurements (from camera images) rather than on calculations.

There were three conditions (Figures 1K–M) that were performed in separate blocks of trials in a random order. Each block of 60 trials was preceded by 10 practice trials that were not analyzed. There were breaks between the blocks while the experimenters gathered the balls. In one condition (*transparent tube*) the balls were visible for 600 ms within the transparent tube and then for about 67 ms after exiting the tube. In a second condition (*early vision*) the lower 1.3 m of the tube was covered, so that the ball was only visible for the first 300 ms of its path through the transparent tube. In a third condition (*late vision*) the top 0.7 m of the tube was covered, so that the ball was only visible for the last 300 ms of its path through the transparent tube.

In this experiment we set the camera so that we could see when the bat started moving forward (by zooming in less). We used this to estimate about how long it took to hit the ball for five trials of each subject in each condition, and determined the overall median of these estimates for each condition. We did not try to determine this time more precisely because it is difficult to tell when exactly the true hitting movement started, but these values provide an indication of the timing of the swing of the bat. Knowing this can help interpret the influence that seeing the ball at different times has on the temporal precision. The target was at a distance of about 5 m and the camera at a distance of about 3.5 m from the ball's path. The further procedure was the same as in Experiment 2. Again, we compared the timing precision across the conditions with a repeated measures analysis of variance, and then used *t*-tests with Bonferroni correction to identify the conditions in which the subjects had significantly different precision.

## Results

### Experiment 1

**Figure 2:**
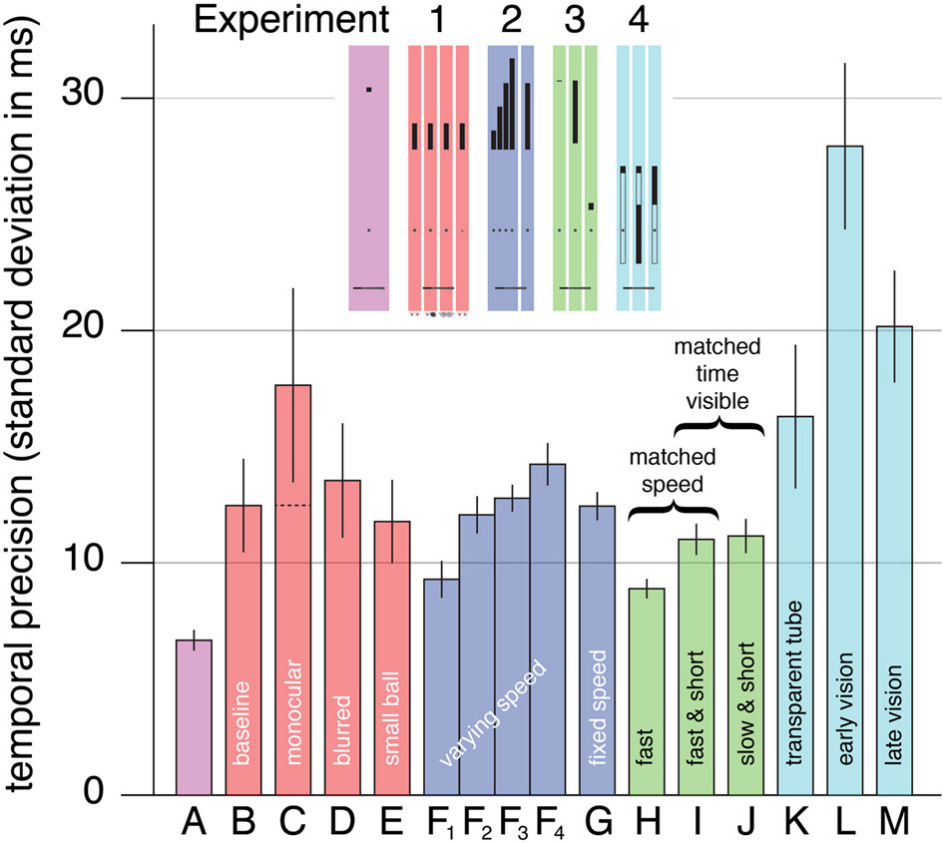
**Mean timing precision**. Each experiment is represented by a different color. The letters indicate the conditions (for a quick graphical reference see the miniature version of Figure 1 that is included as an inset). The leftmost bar (A) shows the corresponding data from Brenner et al. (2012). There are four bars labeled F because there are four release heights in this condition. The dashed line in the bar for the monocular condition of Experiment 1 (bar C) indicates the precision that would be expected by combining two such monocular judgments (that are independent and equally precise). Error bars are standard errors across subjects.

For each subject and condition, the fraction of balls that were touched by the bat before reaching the ground was combined with the motion of the bat to obtain an estimate of the temporal precision (bars **B** to **E** in Figure 2). Even in the *baseline* condition (bar **B**), the temporal precision was considerably poorer than in our previous study (bar **A**). Within the experiment, there were no significant differences between the conditions [*F*_(3, 24)_ = 1.19, *p* = 0.33]. Performance in the *blurred* and *small ball* conditions (bars **D** and **E**) was very similar to the baseline performance, but performance in the *monocular* condition (bar **C**) looks a bit poorer. Although this could be considered to suggest that binocular vision is critical for interception, but that our study does not have enough power to demonstrate this, we do not interpret it in that manner, because closing one eye does not only remove purely binocular information (Rose, 1978; van Mierlo et al., 2011). If the two eyes give independent judgments of the ball's trajectory with a similar resolution, and the two judgments are combined optimally, then using both eyes could lead to an improvement in precision of about a square root of two (Blake et al., 1981; Simpson et al., 2009). The dashed line within bar **C** of Figure 2 indicates the performance that one could expect from optimally combining two independent and equal estimates which each have the precision that we measured for the *monocular* condition. This value is very close to the baseline.

Since neither blurring the image nor using a smaller ball decreased the subjects' temporal precision, it is unlikely that the retinal resolution is critical. That blurring the image did not decrease the subjects' temporal precision also confirms that binocular disparity is unlikely to be critical, because blurring the images can be expected to decrease the resolution for judging distance from binocular disparity (Schor and Wood, 1983; Watt et al., 2005). Not finding any significant differences between the conditions suggests that the ability to estimate the changing angular elevation might be critical, because judging the rapidly changing elevation does not evidently benefit from a sharper or larger retinal image. The resolution with which the angular elevation can be judged is probably limited by the precision with which the orientation of (each of) the eyes is known. On average, the bat was moving at about 18 m/s when it hit (or passed) the ball. The differences between individual subjects' mean hitting speeds in the four conditions (standard deviation of 1 m/s) were much smaller than the differences between the different subjects' hitting speeds within each condition (standard deviation of 5 m/s).

### Experiment 2

Only one trial could not be analyzed because the hit took place outside the image. We estimated the temporal precision for each subject, condition and release height (in the *varying speed* condition; bars **F_1_** to **F_4_** in Figure 2). The critical comparison is between the trials with the same release height in the two conditions (bars **F_3_** and **G**). Precision did not differ between these trials [*F*_(1, 11)_ = 0.44, *p* = 0.52]. Thus, precision for trials with a given release height does not depend on whether or not such trials are interleaved with trials with different release heights. This implies that even in the *fixed speed* condition, subjects were relying on the ball's motion, rather than for instance learning to swing a fixed time after the ball appeared.

Within the *varying speed* condition, precision was lower (the standard deviation was larger) when the ball was moving faster at the time that it appeared [bars **F_1_**–**F_4_**; *F*_(3, 33)_ = 9.53, *p* = 0.00011]. Precision for the lowest speed (bar **F_1_**) was significantly better than for the two highest speeds (bar **F_3_**, *p* = 0.0051; bar **F_4_**, *p* = 0.00022). There was also a tendency to hit later with respect to the ball if the ball was moving faster, but this was not significant. Such a tendency could mean that subjects relied on the velocity on previous trials to some extent (de Lussanet et al., 2001), but it could also just arise because subjects had (too) little time to hit the fastest targets. We estimate that the time between the ball coming into sight and it being hit is about 530, 450, 400, and 370 ms for drops from heights of 4.74, 5.27, 5.79, and 6.34 m, respectively. We intentionally gave subjects so little time to encourage them to use other than visual information.

### Experiment 3

After excluding the subject for whom most trials could not be analyzed because the hit took place outside the image, there were only two additional trials that could not be analyzed. Again, we estimated the temporal precision for each subject and condition (bars **H**, **I**, and **J** in Figure 2). Precision differed significantly between the conditions [*F*_(2, 24)_ = 8.89, *p* = 0.0013]. Subjects timed the hit more precisely in the *fast* condition (bar **H**) than in either the *fast & short* condition (bar **I**; *p* = 0.0055) or the *slow & short* condition (bar **J**; *p* = 0.018). Precision was very similar in the two latter conditions that were matched in terms of the time that the ball was visible. The time that the ball was visible was not exactly the same in both conditions, because subjects hit slightly higher than we had anticipated on the basis of the previous experiments. Consequently, the time that the ball was visible was about 6 ms shorter in the *slow & short* condition than in the *fast & short* condition. Nevertheless, it is evident that the time that the ball is visible is critical, rather than the ball's speed.

### Experiment 4

In this experiment 132 trials could not be analyzed because the hit took place outside the image (8%). We estimated the temporal precision for each subject and condition from the remaining trials (bars **K**–**M** in Figure 2). Precision differed significantly between the conditions [*F*_(2, 16)_ = 22.2, *p* = 0.000024]. Subjects timed the hit more precisely in the *transparent tube* condition (bar **K**) than in the *early vision* condition (bar **L**; *p* = 0.00026). They also timed the hit more precisely in the *late vision* condition (bar **M**) than in the *early vision* condition (*p* = 0.022).

These results show that it is not just better to see the ball earlier, for instance in order to time the onset of the batting movement more precisely (Caljouw et al., 2004; Tresilian and Plooy, 2006). In the *transparent tube* condition, the bat's forward motion took about 240 ms. In the *early vision* condition it took about 300 ms and in the *late vision* condition about 180 ms. That the movement started later and took less time in the *late vision* condition is logical because subjects must wait for the ball to appear before really starting their movement. Why the movements started earlier and took longer in the *early vision* condition is less clear. Perhaps subjects tried to time their hit in relation to the ball disappearing.

The last 67 ms of the ball's motion were always outside the tube (this time did not differ systematically between the conditions). Since it takes at least 100 ms to adjust a movement to new visual information (Brenner and Smeets, 1997; Oostwoud Wijdenes et al., 2011), this does not affect our interpretation. In general, performance was poorer in this experiment than in the previous ones. This is probably because the ball's fall was less consistent within the tube, so that a prediction based on the visible motion within the tube was less accurate than a prediction based on visual motion outside the tube in the other experiments. The precision may also have been affected by it being more difficult to see the ball within the transparent tube. For these reasons, adjusting the on-going swing may have been exceptionally important, and the consequences of doing so therefore exceptionally clear, in this experiment.

## Discussion

In Experiment 1 we examined the three sources of visual information that we considered to most likely underlie, and therefore limit, temporal precision in hitting a falling ball. We found that temporal precision is not limited by the retinal resolution for judging size (as proposed in McLeod and Jenkins, 1991). If that were the case, using a smaller ball or blurring the image (by making subjects near-sighted) would have resulted in poorer precision. If the (non-significant) reduction in precision for monocular viewing is really due to having two, largely independent estimates of the relevant monocular estimate when looking with both eyes, information from ocular convergence and binocular disparity is probably also not critical. This does not mean that retinal expansion and binocular information are not used in interception (Lee et al., 1983; Regan, 1997; Rushton and Wann, 1999). It just means that in our task the critical visual information is probably the changing angular elevation (in this context, it is worth mentioning that a similar lack of sensitivity to blurring the image has also been found for a more conventional batting configuration; Mann et al., 2007, 2010). Even if all three cues are always considered, if one of the three is much more precise than the others, and the three are combined in anything close to an optimal manner, only removing the most precise cue will affect the precision noticeably. If timing in our task is indeed based on changing angular elevation, the results of the monocular condition imply that elevation is judged independently for each eye (considering the orientation of the eye as well as the retinal position of the ball's image) and the two judgments are then averaged.

Experiment 2 rejected an alternative cue, learning to hit at a fixed time after the target appeared, that could have been used in the first experiment and in the previous experiments that found a high temporal precision, because in all those studies targets were dropped from a fixed height. We found that interleaving targets falling from different heights did not make any difference. At the same time, we found that the temporal precision was lower for faster balls. Experiment 3 shows that this is not directly because of the balls' speeds, but because the faster balls were visible for less time (in Experiment 2 they always appeared at the same height, but moving at different speeds). Note that this does not mean that people hit as many of the faster balls as of the slower balls. The time window for hitting the faster balls is shorter, so fewer of the faster balls were hit despite the equal temporal precision. Experiment 4 shows that it is seeing the ball longer that is beneficial, not just seeing it earlier. Thus, visual information is not only used to initiate the swing of the bat at a more precise time, but also to guide the bat during the swing, presumably primarily on the basis of the ball's angular elevation.

By plotting the raw time differences of Experiments 2–4, we confirmed that the timing errors were approximately normally distributed. This justifies the analysis that we used in Experiment 1 and in our previous study (Brenner et al., 2012). To judge how reliably the time differences are determined from the images, we compared repeated estimates for the same trials on different days, by the same person. We found a mean standard deviation of 0.7 ms (averaged across trials). Thus, the contribution of uncertainty in judging the timing from the images is negligible.

In general, performance in this study was slightly worse than in the earlier, comparable studies (McLeod et al., 1985; Brenner et al., 2012). One obvious reason for this is that in many of the current experiments the time for which the ball was visible was quite short. The condition of the current study with the longest time for which the ball was visible before reaching the position at which it was hit is the *fast* condition of Experiment 3 (it was visible for about 970 ms). This is also the condition in which the standard deviation in the timing was smallest. However, even the precision in this condition was poorer than in the former study, in which the ball was visible for 830 ms (Brenner et al., 2012).

The fact that even the best performance was poorer than performance in our former study could be a coincidence, because different subjects took part in the former study and there are considerable differences in precision between subjects, but it is also possible that directly measuring the precision, rather than inferring it from the number of hits, results in a slightly poorer value for the estimate of the precision. There are at least two reasons why measuring the precision directly could give rise to a poorer estimate of the temporal precision. The first reason is that outliers influence the standard deviation that is calculated from individual values considerably, whereas they do not influence the number of hits differently than any other misses. The second possible reason for temporal precision being worse when measuring timing directly, is that we might be overestimating the variability when we calculate the standard deviation of the individual times, because in doing so we implicitly assume that people try to hit in the same way on all trials. We tried to encourage our subjects to do so by asking them to hit a target with the ball, not just to hit the ball. Nevertheless, our subjects may have varied the speed at which they tried to hit the ball, even across identical trials, and therefore intentionally aimed for a slightly different timing relative to the ball on different trials, because if a ball is hit harder it must also be hit later in order to hit the target (see Brenner et al., 2012). A target that is hit earlier and more gently will reach the target along a more curved path.

Considering a preferred duration of the bat's forward motion of about 240 ms (as determined for the *transparent tube* condition of Experiment 4), and a minimal reaction time of about 200 ms (as determined by subtracting the average movement time from the time the ball is visible in the *late vision* condition of Experiment 4; also see Marinovic et al., 2009), we can understand the decrease in precision with ball speed in Experiment 2 (ball visible for about 530, 450, 400, or 370ms before being hit). Assuming that subjects naturally select the optimal movement time for the task, given the prevailing task constraints (Brouwer et al., 2005; Faisal and Wolpert, 2009), the above values indicate that at least for the two fastest ball speeds, subjects will have been forced to move faster than is optimal. However, in Experiment 3, subjects were more precise when the ball was visible for about 970 ms than when it was visible for about 450 ms, suggesting that it is advantageous to see the ball for some time before initiating the forward movement of the bat.

The results of Experiment 4 show that seeing the ball earlier, and therefore having more information with which to select the optimal moment to initiate the swing, is less important than seeing the ball throughout the bat's movement, probably because subjects adjust their bat's motion to that of the ball throughout the movement (Bootsma and van Wieringen, 1990; Peper et al., 1994; Caljouw et al., 2004; Brenner and Smeets, 2011). Taken together, the results of our four experiments suggest that people primarily time their hits so precisely by using the perceived changing elevation of the ball throughout the swing to adjust the bat's movement to that of the ball.

### Conflict of interest statement

The authors declare that the research was conducted in the absence of any commercial or financial relationships that could be construed as a potential conflict of interest.
